# Methodological rigor in climate-resilient health systems research: from criticism to contribution

**DOI:** 10.1186/s12992-023-01002-y

**Published:** 2023-12-14

**Authors:** Ali Mohammad Mosadeghrad, Parvaneh Isfahani, Leila Eslambolchi, Maryam Zahmatkesh, Mahnaz Afshari

**Affiliations:** 1 Health Economics and Management Department, Tehran University of Medical Sciences, Tehran, Islamic Republic of Iran; 2 School of Public Health, Zabol University of Medical Sciences, Zabol, Iran; 3 School of Business and Management, Royal Holloway University of London, Egham, England; 4 School of Nursing and Midwifery, Saveh University of Medical Sciences, Saveh, Iran

**Keywords:** Critical appraisal, Scoping reviews, Systematic reviews

## Dear editor

In our recent article titled “Strategies to strengthen a climate-resilient health system: A scoping review” in the BMC Globalization and Health journal, we embarked on an extensive review of literature, guided by the Arksey and O’Malley scoping review protocol [1]. Through a meticulous search of six diverse databases, we successfully identified 87 strategies to strengthen the climate resilience of the health system. These strategies were organized into the six pillars of health systems: governance and leadership; financing; workforce; medical products, and technologies; information systems; and service delivery. Furthermore, we proposed a conceptual framework to strengthen a climate resilience health system [2].

The editor of BMC Globalization and Health journal informed us about some critical views on our article through a letter to the editor, titled as “Methodological Concerns in the Published Article in Globalization and Health: A Critical Evaluation”. We do value scholarly discourse and constructive feedback, however, here, we found it necessary to reply to the points mentioned in this letter to address the raised methodological concerns.

The first point is that we used Iranian databases in our search strategy which might have affected the comprehensiveness and fairness of the review. It is worth to note that the aim of a scoping review is to map key concepts of a research area rapidly and comprehensively. Therefore, our research was underpinned by an inclusive database selection strategy that incorporated both international (Web of Science, PubMed, Scopus, and EMBASE) and Persian (Scientific Information Database and Magiran) databases, supplemented by Google Scholar search engine. This expansive search strategy was instrumental in uncovering a broader spectrum of solutions to strengthen the health system’s climate resilience than if we had limited our search to English-language databases exclusively. The implication that our study was confined to Iranian contexts is factually incorrect, as illustrated by the geographical distribution of studies presented [2], which spans all six WHO regions.

The second point is the lack of clarity in the methodology regarding eligibility criteria, and data extraction. Again, we draw readers’ attention to the point that our study is a scoping review which is methodologically different from a systematic review. While systematic reviews require the tabulation of each retrieved article’s main characteristics, scoping reviews do not share this obligation. Instead, scoping reviews aim to explore the scope and the breadth of literature, clarify concepts, identify gaps, and inform future research priorities [1, 3], as was our objective. We applied the PRISMA flow diagram to enhance transparency in our data extraction process. This should not be misconstrued as a shift towards systematic review protocols. As such, registration in PROSPERO, a registry for systematic reviews, was not necessary for our scoping review, which adhered to the Arksey and O’Malley protocol [1]. The distinction between the scoping and systematic reviews is paramount (Table 1). Scoping reviews serve to chart the landscape of existing literature on a given topic, and address a broader research question, while systematic reviews delve into answering specific questions, often with a pre-defined scope and stringent quality appraisal of included studies. Our scoping review was a precursor to systematic reviews, setting the stage for subsequent, more focused inquiries.

**Table 1:** Characteristics of scoping reviews and systematic reviews [3]

	Scoping reviews	Systematic reviews
A priori review protocol	Yes (some)	Yes
PROSPERO registration of the review protocol	No	Yes
Explicit, transparent, peer reviewed search strategy	Yes	Yes
Standardized data extraction forms	Yes	Yes
Mandatory Critical Appraisal (Risk of Bias Assessment)	No	Yes
Synthesis of findings from individual studies and the generation of ‘summary’ findings	No	Yes

We stand by the robustness of our methodology and the validity of our findings. We believe that our research provides a valuable contribution to the field and serves as a solid foundation for future studies aimed at strengthening the resilience of health systems in the face of global climate change. In closing, critical appraisal should be a comprehensive examination, encompassing all facets of a study, from its introduction and methodology to its findings, discussion, and conclusion. A critic should engage with the study holistically, and when necessary, directly communicate with the authors to resolve any issues constructively. This ensures that critiques are not only scientifically grounded but also contribute productively to the academic dialogue.

## Data Availability

Not applicable.
